# Treating Depression With Tai Chi: State of the Art and Future Perspectives

**DOI:** 10.3389/fpsyt.2019.00237

**Published:** 2019-04-12

**Authors:** Jian Kong, Georgia Wilson, Joel Park, Kaycie Pereira, Courtney Walpole, Albert Yeung

**Affiliations:** ^1^Psychiatry Department, Massachusetts General Hospital, Harvard Medical School, Charlestown, MA, United States; ^2^Depression Clinical and Research Program, Massachusetts General Hospital, Boston, MA, United States

**Keywords:** Tai Chi, mind–body intervention, major depressive disorder, depression, brain network, anti-inflammation

## Abstract

Major depressive disorder (MDD) is one of the most prevalent mental illnesses in America. Current treatments for MDD are unsatisfactory given high non-response rates, high relapse rates, and undesirable side effects. Accumulating evidence suggests that Tai Chi, a popular mind–body intervention that originated as a martial art, can significantly regulate emotion and relieve the symptoms of mood disorders. In addition, the availability of instructional videos and the development of more simplified and less structured Tai Chi has made it a promising low-intensity mind-body exercise. In this article, we first examine a number of clinical trials that implemented Tai Chi as a treatment for depression. Then, we explore several mechanisms by which Tai Chi may alleviate depressive symptoms, hypothesizing that the intervention may modulate the activity and connectivity of key brain regions involved in mood regulation, reduce neuro-inflammatory sensitization, modulate the autonomic nervous system, and regulate hippocampal neurogenesis. Finally, we discuss common challenges of the intervention and possible ways to address them. Specifically, we pose developing a simplified and tailored Tai Chi protocol for patients with depression, comparatively investigating Tai Chi with other mind–body interventions such as yoga and Baduanjin, and developing new mind–body interventions that merge the advantages of multiple mind–body exercises.

## Introduction

Major depressive disorder (MDD) is a highly prevalent mental illness in the United States (1). Psychotherapy and pharmacotherapy are the mainstay treatments for MDD (2). However, treatment of the disorder is associated with substantial direct and indirect costs, and the clinical efficacy of the treatments has been criticized (3–5). Psychotherapy poses significant time costs on both health professionals and patients, and many patients are troubled by medication side effects such as “sexual dysfunction, weight gain, and sleep disturbance” (6). A significant proportion of patients respond only partially to antidepressants and may require augmentation with other agents in order to enhance the limited effect of the medication (7). Given the disadvantages of first-line treatments, some researchers have begun to explore the effectiveness of alternative therapies.

Tai Chi, a popular mind–body intervention, has recently drawn the attention of the public and researchers alike. Traditional Tai Chi training requires direct supervision from mentors following strict postures. With modern technology and video-sharing sites like Youtube, Tai Chi is becoming increasingly accessible to the general public. We feel it qualifies as a low-intensity exercise, for 1) it can be practiced with video alone and does not require the assistance of a formally trained Tai Chi instructor, and 2) more simplified and less structured Tai Chi has been developed in recent years to accommodate different populations.

Tai Chi employs slow, gentle movements, breathing techniques, and cognitive tools (i.e., attention, imagery) to strengthen, integrate, and relax the body and mind (8). It can be practiced by people of all age groups with varying physical conditions and requires very little physical space. The benefits of Tai Chi on MDD have been supported by a number of well-designed studies (9–11). An advantage of using Tai Chi to treat or augment treatment of MDD is that it is safe and not associated with the adverse events commonly seen with pharmacological agents.

Although promising, Tai Chi’s therapeutic effect needs to be further explored, as the underlying mechanism of the intervention remains unclear. Although different styles of Tai Chi exist, none of these styles have been developed to specifically target patients with depression. The complexity of Tai Chi exercise further inhibits its application in patients with depression. Thus, there is an urgent need to develop a simplified Tai Chi protocol that is tailored for depression.

In this manuscript, we first summarize the findings of clinical studies on Tai Chi treatment of depression. Then, we attempt to summarize the potential mechanisms by which the intervention treats depressive symptoms. Finally, we propose a new direction of Tai Chi research, including a new Tai Chi protocol based on these putative mechanisms. Please also see several recently published review papers on the beneficial effects of Tai Chi for individuals with depression and mood disorders (8, 12, 13).

### Potential of Tai Chi Treatment of Depression—Results From Clinical Studies

In an earlier study, Chou (14) investigated the effects of Tai Chi on the depressive symptoms of 14 older Chinese patients. Researchers found that 3 months of Tai Chi intervention can significantly reduce scores on the Center for Epidemiological Studies Depression Scale (CES-D) and all of its subscales (including symptoms related to somatic, negative affect, interpersonal relations, and well-being) as compared to a waitlist control. These decreased scores remained significant after controlling for age, gender, and education but not after controlling for social support changes, as measured by the Lubben Social Network Scale (LSNS). This finding suggests that social support may contribute to the effect of Tai Chi on depressive symptoms. This was one of the earliest studies investigating the effect of Tai Chi on depression with positive and notable findings. However, it was limited by a small sample size and the use of a passive control.

In a follow-up study, Lavretsky et al. (15) explored if SSRI (escitalopram) treatment supplemented with 10 weeks of Tai Chi, as compared to health education (HE), would enhance depression treatment in 73 older adults. They found that patients in the Tai Chi-supplemented condition were more likely to 1) experience a greater improvement in depressive symptoms or achieve depression remission and 2) have greater improvements in C-reactive protein levels and the 36-Item Short Form Health Survey physical functioning and cognitive tests compared to the control group. These findings suggest that supplementing pharmacologic treatment with Tai Chi may yield greater clinical improvement for individuals with geriatric depression. This study had a large sample size and obtained positive findings in both patients’ subjective ratings and in inflammatory marker levels. It demonstrates the benefits of adding Tai Chi to an antidepressant regimen but does not examine the specific effect of Tai Chi on depression.

Field et al. (16) investigated the effects of combined Tai Chi/yoga in 92 prenatally depressed pregnant women. They found that women practicing Tai Chi/yoga (20 min per week for 12 weeks) had lower depression, anxiety, and sleep disturbance scores compared to a waitlist control group (**Table 1**). This study had a large sample size and provided important evidence on the effects of Tai Chi on depressed pregnant women, who generally would avoid pharmacologic treatment. However, combining Tai Chi and yoga is uncommon in the real world, and a waitlist is considered a weak control.

**Table 1:** Tai Chi studies applied on patients with depression and primary outcomes.

Study	Patient population demographics/sample size	Setting	Treatment (*n*)/control (*n*)	Treatment-related information	Primary outcome measures	Major results
Yeung et al. (10)	39 Chinese Americans with MDD; 77% women; mean (SD) age = 55 (10)	Group Tai Chi class in Boston, MA; taught in Chinese (Cantonese, Mandarin)	Tai Chi: *n* = 26 Waitlist: *n* = 13	Tai Chi: 1-h class twice a week, 12 weeks Waitlist: 12 weeks	Depression severity following treatment, as measured by the HAM-D_17_	Response and remission rates were better in the Tai Chi group versus the waitlist group. However, these differences were not significant (*p* = 0.15 and *p* = 0.30 for response and remission rates, respectively). Tai Chi was proven safe and feasible for Chinese Americans.
Chou (14)	14 Chinese patients with MDD, ages 60 and older	Classes led by Tai Chi instructor in group setting	Tai Chi: *n* = 7 Waitlist *n* = 7	Tai Chi: 3 × 45 min/week, 12 weeks Waitlist: 12 weeks	Depression severity following treatment, as measured by the CES-D	Tai Chi can yield a reduction in depressive symptoms compared to a waitlist control (main effect of group assignment: 0.82, *p* < 0.01). Controlling for social support between groups removes any benefit of Tai Chi on CES-D scores. Thus, social support may contribute to the effects of Tai Chi on depressive symptoms.
Lavretsky et al. (15)	73 adults over 60 with MDD; Escitalopram with Tai Chi group: 64% women, mean (SD) age = 69.1 (7.0); Escitalopram with health education (HE) group: 60% women, mean (SD) age = 72.0 (7.4)	Both Tai Chi and HE classes were conducted by study staff. Tai Chi classes included a warm up and cool down. HE classes included lectures and discussion.	Escitalopram with Tai Chi: *n* = 36 Escitalopram with HE: *n* = 37	Escitalopram with Tai Chi: 2 h/week, 10 weeks Escitalopram with HE: 2 h/week, 10 weeks	Depression severity following treatment, as measured by the HAM-D_24_; subjects were classified as “remission,” “response,” and “nonresponse” according to score	A higher percentage of participants achieving remission and response was observed in the escitalopram with Tai Chi group than in the escitalopram with HE group (*p* < 0.05). Compared to HE, Tai Chi may better augment the effects of SSRI medication in the treatment of major depression.
Field et al. (16)	92 pregnant women with MDD; mean (SD) age = 26.6 (5.5); range = 18–37	Participants recruited from medical clinic; classes taught in group setting	Tai Chi with Yoga: *n* = 46 Waitlist: *n* = 46	Tai Chi with yoga: 20 min/week, 12 weeks Waitlist: 12 weeks (received Tai Chi/yoga intervention after initial 12 weeks)	Depression severity following treatment, as measured by the CES-D	Tai Chi with yoga intervention yielded a greater reduction in depressive symptoms than the waitlist control (*p* = 0.001).
Yeung et al. (11)	67 Chinese Americans with MDD; ages 18 to 70	Group Tai Chi class in Boston, MA; taught in Chinese (Cantonese, Mandarin)	Tai Chi with yoga: *n* = 23 Waitlist: *n* = 22 Healthy education: *n* = 22	Tai Chi: 1-h class twice a week, 12 weeks Waitlist: 12 weeks Healthy education: 1-h class twice a week, 12 weeks	Response and remission rates following treatment, as measured by HDRS_17_	Tai Chi intervention yielded greater response and remission rates than the waitlist group (odds ratio for response = 2.11, 95% CI; odds ratio for remission = 3.01, 95% CI). Tai Chi intervention yielded a significantly greater response rate (odds ratio = 8.90, 95% CI) but an insignificantly greater remission rate (odds ratio = 4.40, 95% CI) compared to HE group.

MDD, major depressive disorder; HE, health education; CES-D, Center for Epidemiological Studies–Depression Scale; HAM-D_17_/HDRS_17_, Hamilton Rating Scale for Depression (17 items); HAM-D_24_, Hamilton Rating Scale for Depression (24 items); CI, confidence interval.

In another study, Yeung and colleagues (10) examined the practicality and outcome of using Tai Chi to treat depressive symptoms in 39 Chinese Americans with MDD. They found that 73% of patients in the Tai Chi group completed the intervention, and no adverse events were reported. This was a proof-of-concept study with a small sample size to investigate the feasibility and safety of Tai Chi for depressed Chinese Americans, a population that tends to avoid conventional mental health services due to their high levels of stigma against having mental illnesses. In a following study with a larger sample size (*n* = 67) (11), researchers found that following a 12-week intervention, “response rates were 25%, 21%, and 56%, and remission rates were 10%, 21%, and 50% for the waitlisted, education, and Tai Chi intervention groups, respectively.” Participants randomized to the Tai Chi group experienced a greater response to treatment than did individuals randomized to the waitlist and education groups. Further, participants in the Tai Chi group experienced a significantly greater remission rate than the waitlist group and “a trend of improved remission compared to the education group” (**Table 1**). This study provided preliminary evidence on the effectiveness of Tai Chi on depressed Chinese Americans, using both passive and active control groups. When the Tai Chi group was compared to the passive control group, improvements in both response rate and remission rate showed statistical significance. Yet, when the Tai Chi group was compared to the active control group, improvement in response rate showed statistical significance but not for improvement in remission rate. The relatively small sample in each of the study groups may explain this negative finding. Studies with larger sample sizes will be needed to provide a more definitive conclusion.

Depression is also a common disorder among elderly adults, and many studies have explored depression treatment for this population (17, 18). Brown et al. (19), for instance, compared psychological changes associated with 16 weeks of moderate-intensity walking (MW), low-intensity walking (LW), low-intensity walking plus relaxation response (LWR), and Tai Chi in healthy, sedentary adults. They found that women in the Tai Chi group experienced decreased mood disturbance (tension, depression, anger, confusion, and total mood disturbance) and an improvement in general mood. Women randomized to the MW group also reported increased satisfaction with physical attributes (body cathexis), while men in the same group reported increased positive affect. These results suggest that mind–body interventions such as Tai Chi may have more psychological benefits than exercises without a cognitive component, thus demonstrating Tai Chi’s value in promoting mental health.

Finally, there is accumulating evidence that Tai Chi can relieve depressive symptoms in patients with fibromyalgia (20, 21), arthritis (22–24), multiple sclerosis (MS) (25), heart failure (26–28), mild dementia (29), and cerebrovascular disorder (30).

### Mechanisms of Tai Chi Treatment of Depression

#### Tai Chi Can Modulate the Brain Regions/Networks Associated With Depression

Studies have shown that depression is linked to structural and functional abnormalities in brain regions that are associated with emotion processing, self-representation, reward, and external stimulus (i.e., stress, distress) interactions (31–39). Among these brain regions are the hippocampus, amygdala, anterior cingulate, ventromedial prefrontal cortex, and dorsomedial prefrontal cortex.

**Figure 1:**
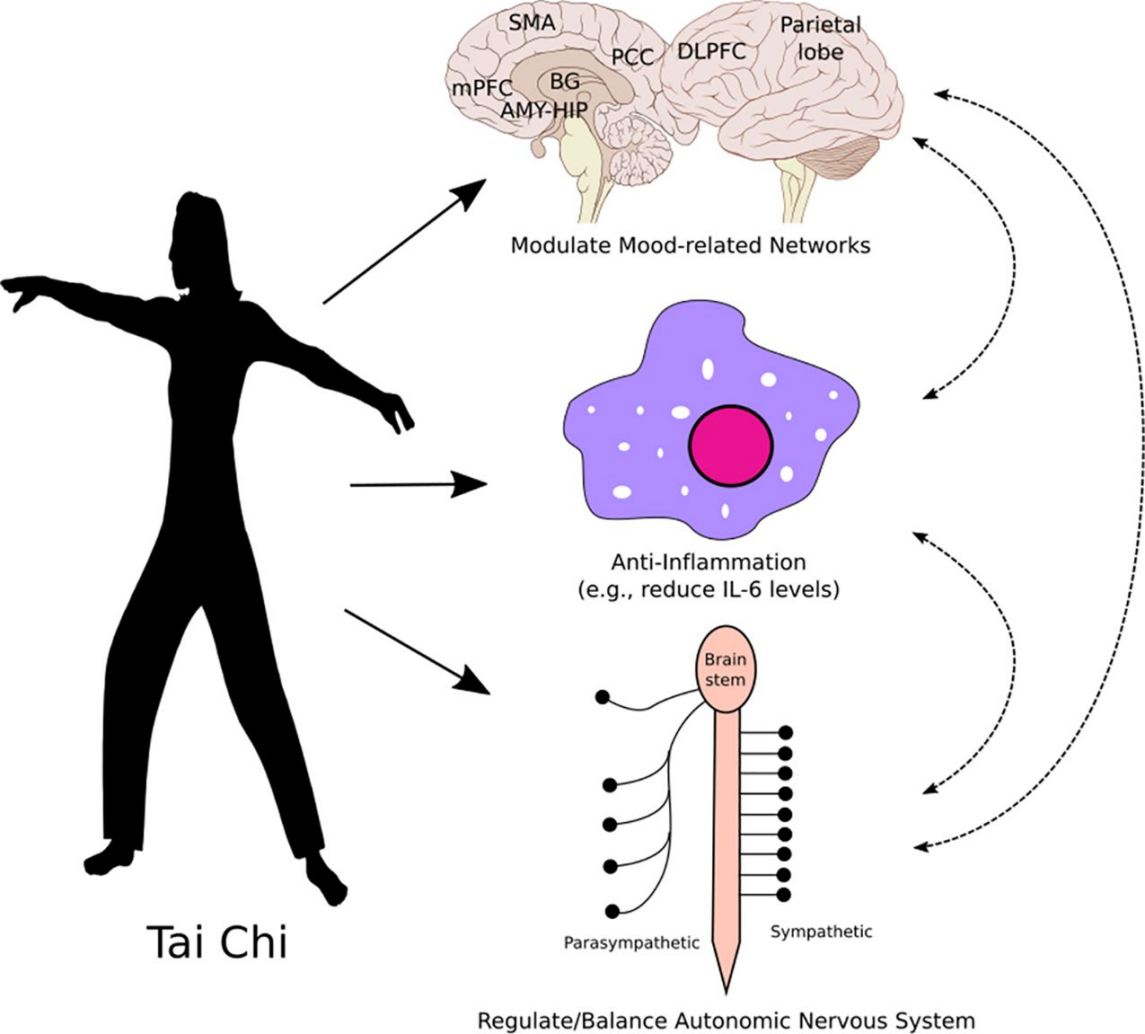
Hypothesized mechanisms of Tai Chi treatment of depression: directly and indirectly modulating the activity and connectivity of key brain regions involved in depression and mood regulation, reducing neuro-inflammatory sensitization, and modulating the autonomic nervous system. Abbreviations: AMY, amygdala; BG, basal ganglia; DLPFC, dorsolateral prefrontal cortex; HIP, hippocampus; mPFC, medial prefrontal cortex; PCC, posterior cingulate cortex; SMA, supplementary motor area.

Studies also suggest that core components of mind–body interventions such as Tai Chi may include attentional control, emotion regulation, and self-awareness (40). Although no brain-imaging study has directly investigated the modulation effect of Tai Chi in patients with depression, brain-imaging studies in healthy subjects and other patient populations have endorsed the potential pathways of Tai Chi modulation. Furthermore, results from intervention studies support that the target brain regions outlined in this manuscript can indeed be altered by various interventions in patients with depression (**Figure 1**). For instance, a systematic review by Gudayol-Ferre et al. notes that “antidepressive treatment is capable of normalizing brain activations in depressed patients during affective tasks in areas such as the DLPFC,” and treatments for depression are associated with alterations in the default mode network (DMN) (41).

One potential mechanism of Tai Chi on MDD may occur *via* the cognitive (attentional) control network (40). During Tai Chi, attention is focused on body posture/movement and breathing regulation and is shifted away from the stressor. Thus, repeated Tai Chi exercise (movement in a mindful way) can modulate the attentional control network and relieve depressive symptoms, and noradrenaline may be heavily involved in attention regulation (42).

In support of this hypothesis, we found that Tai Chi can significantly modulate resting state functional connectivity (rsFC) of the dorsolateral prefrontal cortex (DLPFC), a key region of the cognitive (attentional) control network, with the medial prefrontal cortex and anterior cingulate cortex (MPFC/ACC), key regions of the DMN and limbic system, in healthy elderly adults (43) and patients with fibromyalgia (44). In another study, Wei et al. found that Tai Chi may decrease fractional amplitude of low-frequency fluctuations (fALFF) in the bilateral frontoparietal network (executive/attentional control network). Researchers also identified an association between cognitive control performance and fALFF of the frontoparietal network (45). In a more recent study (46), investigators found that Tai Chi can significantly decrease the functional connectivity between the DLPFC and the thalamus, ventral striatum, and right middle frontal gyrus compared to the control group.

Studies suggest that Tai Chi can also modulate the DMN, a brain network involved in self-referential processing, affective cognition, and emotion regulation (47–54). For instance, results from a previous study revealed significantly decreased fALFF in the Tai Chi condition compared to a control, as well as a significant association between mind–body practice experience and fALFF in the DMN (45). As mentioned above, we also found that Tai Chi can modulate functional connectivity between the cognitive control network and key regions of the DMN (MPFC/ACC), indicating that Tai Chi can modulate the interaction of the two networks. One of the most reliable brain-imaging findings on MDD is the disruption of the DMN (55–61). Thus, Tai Chi may also relieve depressive symptoms by modulating the DMN. In addition, studies have shown that Tai Chi can significantly modulate brain structures in areas related to mood regulation, such as the insula, putamen, and medial temporal pole (62, 63). Taken together, the above findings suggest that the central nervous system may play an important role in the modulation effect of Tai Chi.

#### Tai Chi may Relieve Symptoms of Depression by Reducing Stress and Modulating the Inflammation System

Stress is produced as the brain and body respond to demands. Literature suggests that stress, particularly long-term stress, may initiate cognitive, affective, and biological processes that increase one’s risk for depression (64, 65) and that inflam­mation may be involved in this process. Specifically, stress-provoked neuro-inflammatory sensitization can lead to signif­icant be­havioral changes and the onset of common depressive symptoms, such as sad mood, anhedonia, fatigue, psychomotor retardation, and social–behavioral withdrawal (64–67). The hypothalamus, anterior insula, and ACC may be involved in this process (64).

Recent studies have suggested that mind–body interventions like Tai Chi may reduce stress and modulate the inflammation process (68, 69) (**Figure 1**). For instance, Jin (70) found that Tai Chi can raise heart rate, increase noradrenaline excretion in urine, and decrease salivary cortisol concentrations. Compared to baseline levels, subjects reported less tension, depression, anger, fatigue, confusion, and anxiety and felt more vigorous. Irwin and Olmstead (71) evaluated the effects of Tai Chi on circulating markers of inflammation in older adults and found that, among those with elevated Interleukin 6 (IL-6) at entry, Tai Chi yielded a reduction in IL-6 levels comparable to those found in Tai Chi and HE subgroups who had low levels of IL-6 at entry. Meanwhile, IL-6 in HE subgroups remained higher than the Tai Chi and HE subgroups with low IL-6 at entry. Depressive symptom decreases in the two treatment groups were correlated with IL-6 decreases.

Finally, Tai Chi is a mild to moderate exercise, and many studies (72, 73) have suggested that physical exercise itself can modulate the immune and inflammation systems. Such findings endorse the role of Tai Chi in reducing stress and producing anti-inflammatory effects.

#### Other Potential Mechanisms

Previous studies have suggested that MDD is associated with decreased activity of the parasympathetic nervous system (74–77). Heart rate variability (HRV) is a noninvasive index for monitoring the dynamic equilibrium between sympathetic and parasympathetic nervous system activity. Studies have shown that the high-frequency (HF)–HRV component is inversely correlated with depression severity and a marker of treatment response (75, 78–80).

Lu and Kuo (81) found that Tai Chi can yield increased vagal modulation and shift the sympathovagal balance towards decreased sympathetic modulation in elderly individuals. Audette and colleagues (82) compared the effects of a shortened Tai Chi regimen and a brisk walking training program on metrics such as aerobic capacity, HRV, and strength in elderly sedentary women, and researchers found a significant improvement in estimated VO2 max in the Tai Chi group. In the Tai Chi group only, the mean within-person change of HF power in normalized units was increased, representing increased parasympathetic activity. Meanwhile, low-frequency (LF) power in normalized units (nu) decreased, representing decreased sympathetic activity. In a recent meta-analysis on 17 randomized controlled trials (RCTs), investigators (83) found that combined Tai Chi/yoga produced significantly beneficial effects on HRV parameters (normalized LF, normalized HF, and LF-to-HF ratio) and stress level. These results suggest that the autonomic nervous system may also be involved in the modulation effects of Tai Chi on depressive symptoms (**Figure 1**).

According to the neurogenic theory (84), depression results from impaired adult hippocampal neurogenesis, the restoration of which leads to recovery. Studies have shown that Tai Chi can significantly modulate the gray matter volume of the hippocampus (63) and rsFC of the hippocampus with the medial prefrontal cortex, a key region of the DMN (85). These findings imply that Tai Chi may alter hippocampal neurogenesis to reduce depressive symptoms (86). The various mechanisms by which Tai Chi may improve depressive symptoms is illustrated in **Figure 1**.

### Challenges and Future Directions

#### Simplification of Tai Chi Protocol

The complexity of some Tai Chi movements has significantly limited its application as a clinical intervention. Also, not all elements of Tai Chi are relevant to mood regulation and the maintenance of health. Thus, there is a pressing need to develop simplified Tai Chi protocols.

The simplification of Tai Chi has recently begun in China, its country of origin. An example of such simplification is the eight-style Tai Chi that is considered a part of the Chinese martial arts system. Eight-style Tai Chi consists of 10 total postures, including the beginning, the end, and eight main actions from Yang-style Tai Chi.

One attempt to simplify Tai Chi in the Western world is the 12-week Tai Chi protocol developed by Dr. Peter Wayne. The first section introduces several traditional Tai Chi warm-up exercises that can last 15–30 min. The second section focuses on five core Tai Chi movements following the Cheng Ma Ching Yang-style short form. Participants progressively add these movements over the course of the 12 weeks. The program concludes with 5 min of simple cool-down exercises. The total exercise lasts about 45–60 min (87).

Given the ease of the simplified Tai Chi protocols, novice students may practice Tai Chi by following the instructors’ movements in class or at home using videotaped clips. It is worth noting that although these simplified protocols are encouraging, how they differ from traditional Tai Chi remains unknown. Comparative studies are needed to compare the beneficial effects of traditional Tai Chi and simplified Tai Chi.

#### How Tai Chi Differs From Other Mind–Body Exercises

Mind–body exercise encompasses a family of complex practices such as Tai Chi, yoga, and Baduanjin, each with different characteristics and foci. Although the exact mechanisms of these interventions are still under investigation, studies have found that the underlying mechanisms of different mindfulness movements may not be identical (88, 89).

In previous studies, we compared the modulation effect of Tai Chi and Baduanjin, another mind–body exercise. Baduanjin consists of only eight postures; thus, it is much simpler than other mind–body exercises and can be easily practiced by older adults, specifically those with cognitive decline, when guided by video or audio at home. As a result, we found that Baduanjin can produce greater and more extensive improvements in Wechsler Memory Scale (WMS) subscores and gray matter brain volume changes than Tai Chi in healthy older adults (63).

In another study (90), we compared the modulation effect of Tai Chi with Baduanjin on the DMN. We found that compared to the control group, Tai Chi increased posterior cingulate cortex rsFC with the right putamen/caudate, while Baduanjin decreased rsFC between the mPFC and orbital prefrontal gyrus/putamen. Direct comparison between the two mind–body interventions revealed that Tai Chi was significantly associated with increased rsFC between the mPFC and right putamen/caudate compared to Baduanjin, suggesting that these interventions may be associated with different mechanisms and treatment effects.

#### Developing a Tailored Tai Chi Protocol for Different Disorders

One important direction of Tai Chi research may be to create a tailored Tai Chi regimen for different disorders and individuals. Specifying a Tai Chi protocol for a particular population may significantly enhance the benefits of Tai Chi and reduce potential adverse effects.

In one such attempt, Tai Chi practitioner and researcher Dr. Albert Yeung developed a Tai Chi protocol specifically for individuals with mood disorders: Tai Chi for Mood (Tai Chi^M^). The Tai Chi^M^ protocol consists of 12 separate Tai Chi moves along with mental focus and deep, paced breathing. Tai Chi^M^ excludes the complex transitions between Tai Chi movements, making it easier to learn and potentially a stronger intervention. A unique characteristic of Tai Chi^M^ is that it focuses more on meditative movements, relaxation, and paced breathing and less on physical strength training. Such characteristics make it an optimal choice for those with MDD and other mood disorders.

While furthering the development of Tai Chi may be enhanced with tailored protocols, this area of research also demands more fully powered RCTs. The studies summarized in this manuscript support the efficacy of Tai Chi in improving depressive symptoms, but future studies should strive to increase their sample sizes. Conducting studies with larger and more diverse samples will allow us to test the effects of Tai Chi on different subgroups of the MDD patient population. In addition, most of the current studies have been performed on an Asian population, and future studies targeting other populations are needed. Finally, future research should examine the differences between traditional Tai Chi and simplified, low-intensity Tai Chi to identify the most crucial components of this promising mind–body intervention. For instance, it is worth investigating which elements of traditional Tai Chi can be excluded without sacrificing the efficacy of the practice. We believe that the results obtained will facilitate the acceptance of Tai Chi and lower the cost of the practice, thereby making it a more economical and accessible alternative to pharmacologic and other treatments for depression.

We believe that Tai Chi^M^ could be a potential solution to the shortage of mental health providers and well-trained Tai Chi instructors worldwide. Further, it could reduce disparities in mental health treatment among ethnic minority populations who tend to avoid conventional psychiatric treatment due to stigmas surrounding mental illness. To enhance its availability, a video demonstrating Tai Chi for mood is publicly available on YouTube with Chinese, English, and Spanish subtitles (https://youtu.be/08IFKiXb3bA).

In summary, although Tai Chi has demonstrated its potential in mood regulation and relieving depressive symptoms, its underlying mechanism of action remains to be discovered. The instructional videos and the development of less structured Tai Chi has made it a promising low-intensity mind-body therapy. A fully powered RCT would largely benefit the development of Tai Chi, as would an exploration of ways to lower the cost of the intervention. Most importantly, a simplified and tailored Tai Chi—or a new intervention that combines Tai Chi and other mind–body exercises to enhance modulation effects on a specific patient population or individual—may represent a direction for further development of modern Tai Chi.

## Author Contributions

JK and AY conceived of the ideas presented in this manuscript. All authors contributed to manuscript preparation.

## Funding

JK is supported by R01 AT008563, R33AT009310, and R21AT008707 from NIH/NCCIH.

## Conflict of Interest Statement

JK holds equity in a startup company (MNT) and a pending patent to develop a new neuromodulation device.

The remaining authors declare that the research was conducted in the absence of any commercial or financial relationships that could be construed as a potential conflict of interest.
